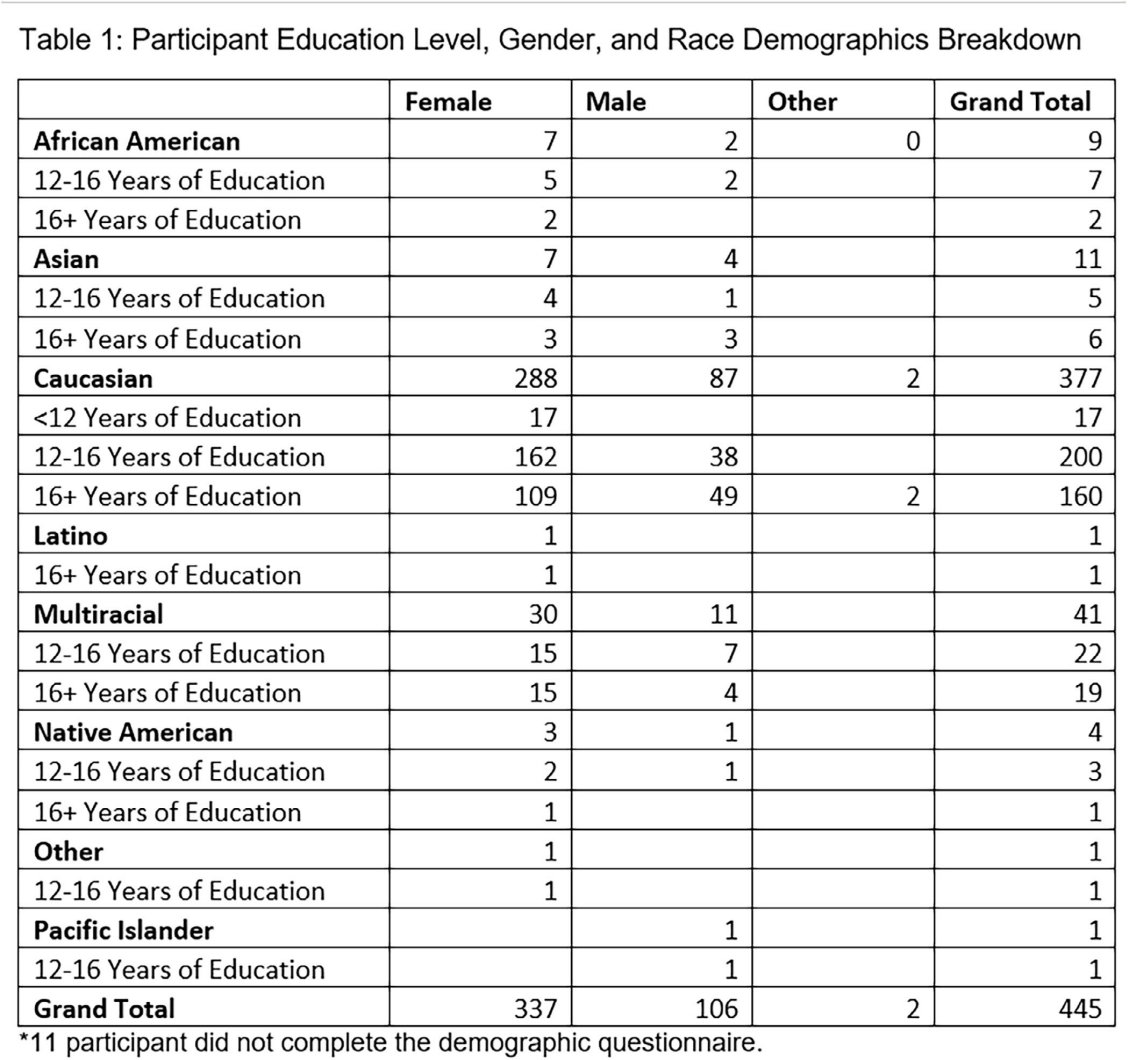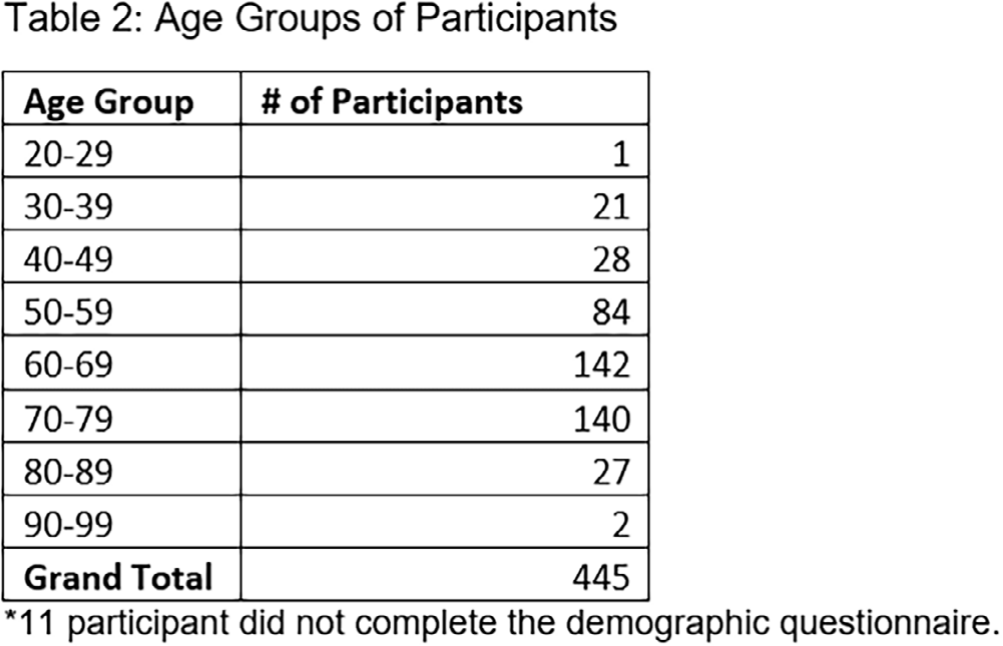# Subjective cognitive concerns are associated with unsupervised smartphone‐based measures of memory and executive functioning in cognitively intact adults

**DOI:** 10.1002/alz.093471

**Published:** 2025-01-09

**Authors:** Sreya Dhanam, Clayton Young, Mai Anh Bui, Jack C. Taylor, Amy B. Wise, Mark E. Sanderson‐Cimino, Emily W. Paolillo, Annie L Clark, Hilary W. Heuer, Winnie Kwang, Joel H. Kramer, Brad F. Boeve, Howard J. Rosen, Michael W Weiner, Rachel L. Nosheny, Adam M. Staffaroni, Adam L. Boxer

**Affiliations:** ^1^ Memory and Aging Center, UCSF Weill Institute for Neurosciences, University of California, San Francisco, San Francisco, CA USA; ^2^ UCSF Memory and Aging Center, San Francisco, CA USA; ^3^ University of California San Francisco (UCSF), San Francisco, CA USA; ^4^ Memory and Aging Center, UCSF Weill Institute for Neurosciences, San Francisco, CA USA; ^5^ San Francisco Veterans Affairs Medical Center, San Francisco, CA USA; ^6^ Department of Neurology, Mayo Clinic, Rochester, MN USA; ^7^ UCSF Weill Institute for Neurosciences, University of California, San Francisco, CA USA; ^8^ San Francisco Veterans Administration Medical Center (SFVAMC), San Francisco, CA, CA USA

## Abstract

**Background:**

Subjective cognitive concerns are common in functionally intact adults, potentially indicating future cognitive decline. Remote smartphone cognitive testing holds promise for objectively tracking cognition in individuals reporting complaints. In our initial exploration of the link between subjective cognitive complaints and digital clinical outcomes, we examined participants' self‐reported cognitive complaints' association with smartphone tests on memory and executive functioning.

**Methods:**

Participants, without self‐reported MCI or dementia, were recruited from the UCSF Brain Healthy Registry (BHR) to complete cognitive tasks on their personal smartphone with the ALLFTD Mobile App. Participants also completed the 12‐item Everyday Cognition Screening Tool (ECog‐12), assessing subjective cognitive impairment, online as part of BHR. Average ECog‐12 scores, including Memory and Executive Functioning subdomains, were calculated. Correlations between scores from online ECog‐12 scores and app‐based smartphone tasks were examined. Regression analyses, adjusting for age, education, and sex, were performed to evaluate the relationship between ECog‐12 and smartphone tasks (Stroop & Flanker) measuring executive function and memory.

**Results:**

Out of 2,283 invited BHR participants, 456 (mean age: 64.6 years (SD=11.8) and education: 16.3 years (SD=2.4)) completed smartphone tasks (81.1% completion rate). Age was negatively associated with executive functioning and memory tasks (β’s=‐0.51 to ‐0.54, p’s<0.05), while higher education correlated with better performance (β’s=0.10‐0.14, p’s<0.05). Sex was not significantly associated with performance on any task (β’s=‐0.04 to 0.09, p’s>0.05). Subjective cognitive concerns were linked to tasks of executive functioning (Stroop: β=‐0.18, p=0.002) and memory (β=‐0.14, p=0.02). Endorsement of executive functioning items on ECog‐12 associated with Stroop performance (β=‐0.16, p=0.005), and memory‐specific complaints were related to memory performance (β=‐0.1, p=0.09). The relationship with flanker scores was not significant (β=‐0.08, p=0.17). Similar patterns emerged with the online ECog‐12; online and app‐based scores are strongly correlated (β=0.75, p<0.05).

**Conclusions:**

In this cognitively intact sample, subjective cognitive concerns were linked to poorer performance on ALLFTD Mobile App tasks assessing executive functioning and memory. These findings suggest that unsupervised remote smartphone testing merits further consideration to monitor cognition in individuals with subjective cognitive concerns. Subsequent studies in deeply phenotyped cohorts with follow‐up assessments are essential for understanding the clinical relevance of these results.